# Bi Nanospheres Embedded in N‐Doped Carbon Nanowires Facilitate Ultrafast and Ultrastable Sodium Storage

**DOI:** 10.1002/advs.202401730

**Published:** 2024-05-02

**Authors:** Qian Yao, Cheng Zheng, Kejun Liu, Mingyue Wang, Jinmei Song, Lifeng Cui, Di Huang, Nana Wang, Shi Xue Dou, Zhongchao Bai, Jian Yang

**Affiliations:** ^1^ Key Laboratory of Colloid and Interface Chemistry Ministry of Education School of Chemistry and Chemical Engineering Shandong University Jinan 250100 P. R. China; ^2^ Department of Biomedical Engineering Research Center for Nano‐Biomaterials & Regenerative Medicine College of Biomedical Engineering Taiyuan University of Technology Taiyuan 030024 P. R. China; ^3^ Institute for Superconducting and Electronic Materials University of Wollongong Innovation Campus, Squires Way Wollongong NSW 2500 Australia; ^4^ Shandong Hualu‐Hengsheng Chemical Co., Ltd. Dezhou 253024 P. R. China; ^5^ Institute of Energy Materials Science (IEMS) University of Shanghai for Science and Technology Shanghai 200093 P. R. China

**Keywords:** Bi, full cell, N‐doped carbon nanowires, rate capacity

## Abstract

Sodium ion batteries (SIBs) are considered as the ideal candidates for the next generation of electrochemical energy storage devices. The major challenges of anode lie in poor cycling stability and the sluggish kinetics attributed to the inherent large Na^+^ size. In this work, Bi nanosphere encapsulated in N‐doped carbon nanowires (Bi@N‐C) is assembled by facile electrospinning and carbonization. N‐doped carbon mitigates the structure stress/strain during alloying/dealloying, optimizes the ionic/electronic diffusion, and provides fast electron transfer and structural stability. Due to the excellent structure, Bi@N‐C shows excellent Na storage performance in SIBs in terms of good cycling stability and rate capacity in half cells and full cells. The fundamental mechanism of the outstanding electrochemical performance of Bi@N‐C has been demonstrated through synchrotron in‐situ XRD, atomic force microscopy, ex‐situ scanning electron microscopy (SEM) and density functional theory (DFT) calculation. Importantly, a deeper understanding of the underlying reasons of the performance improvement is elucidated, which is vital for providing the theoretical basis for application of SIBs.

## Graphical abstract

Bi nanoparticles encapsulated in N‐doped carbon nanowires (Bi@N‐C) are fabricated via a sample electrospinning and carbonization. N‐doped carbon mitigates the structure stress/strain upon alloying/dealloying, optimizes the ionic/electronic diffusion, and provides fast electron transfer and structural stability. Benefiting from the structure, Bi@N‐C shows excellent cycling and rate capacity in half cells and full cells, which is vital for the practical application of Bi‐based for SIBs.

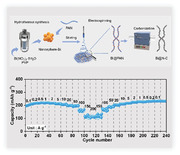

## 1 Introduction

Sodium‐ion batteries (SIBs) are considered as the most promising candidates for the large‐scale energy storage systems because of the natural abundance of sodium reserves.^[^ ^1^ ^]^ However, graphite anode for commercial lithium‐ion batteries (LIBs) shows deficient sodium storage due to the large radius of sodium ions (1.02 Å) compared to lithium ions (0.72 Å).^[^ ^2^ ^]^ Therefore, the major challenge is largely short of ideal anode materials with high energy density and stable cycling stability makes to construct high‐performance SIBs.

The ideal anode materials are expected to possess high capacity, excellent rate capacity, satisfactory cycling‐life, and moderate voltage platform.^[^ ^3^ ^]^ Therefore, alloy‐based anodes have attracted great attention due to their suitable operating voltage, high energy density, and great electrical conductivity.^[^ ^4^ ^]^ Among them, Bismuth (Bi) is considered as a potential candidate for high‐rate electrodes due to its high electronic conductivity, the appropriate operating voltage (≈0.6 V vs Na^+^/Na), and gravimetric specific capacity of 386 mAh g^−1^ based on the product of the alloying reaction of Na_3_Bi.^[^ ^5^ ^]^ However, the huge volume change of Bi during the alloying/dealloying process (≈244%) leads to the unstable solid electrolyte interphase (SEI) and capacity fade, limiting its practical application in SIBs.^[^ ^6^ ^]^ Enormous attention has been devoted to solving these problems of Bi, such as size controlling to nanometers,^[^ ^7^ ^]^ coupling with ether‐based electrolyte,^[^ ^8^ ^]^ preparation of porous structure,^[^ ^9^ ^]^ and carbon coating/encapsulation.^[^ ^10^ ^]^ However, the cycling and rate performance of Bi‐based anode materials can be further optimized. In order to obtain the capacity and rate performance of the anode materials, it is necessary to adopt a reasonable structural design. Therefore, the goals of designing the well‐balanced composite structure are not only to enhance the cycling performance and rate capacity of half cells, but more importantly, to attain excellent rate performance and stable cycling performance of full cells.

Here, we present a proper design and fabrication of a hybrid by encapsulating Bi nanoparticles in N‐doped carbon nanowires (Bi@N‐C) via sample electrospinning. The interwoven carbon framework derived from polyacrylonitrile (PAN) not only possesses high electrical conductivity and enables rapid electron transmission, but also exhibits high porosity and a large surface area, facilitating the thorough penetration of electrolytes. In addition, the N‐doped carbon increases the electrochemical active sites and shortens the electrons/ions diffusion length, while the numerous Bi nanoparticles enhance the rate of utilization of active materials. Consequently, Bi@N‐C shows an ultra‐long cycling stability (197.7 mAh g^−1^ after 4000 cycles at 10 A g^−1^) and excellent rate capacity (110 mAh g^−1^ at 200 A g^−1^). Even at −20 °C, Bi@N‐C can deliver a high capacity of 199.2 mAh g^−1^ at 1 A g^−1^ after 2000 cycles. When Bi@N‐C coupled with homemade NVP@C, the full cell can deliver a capacity of 136.1 mAh g^−1^ after 1000 cycles at 10 A g^−1^ and 161.0 mAh g^−1^ even at a high current density of 20 A g^−1^. Moreover, we deeply investigate the structural evolution of Bi@N‐C during the alloying/dealloying process by atomic force microscopy (AFM), ex situ scanning electron microscopy (SEM), synchrotron in‐situ XRD, and density functional theory (DFT) calculation. It provides a comprehensive understanding of the kinetic optimization, fast electron transfer, mechanical stability, and cycling/rate stability of Bi@N‐C electrodes during the charging and discharging processes. The electrochemical performance of Bi@N‐C||NVP@C full cell is superior to the case of the reported works.

## 2 Results and Discussion

**Figure 1:**
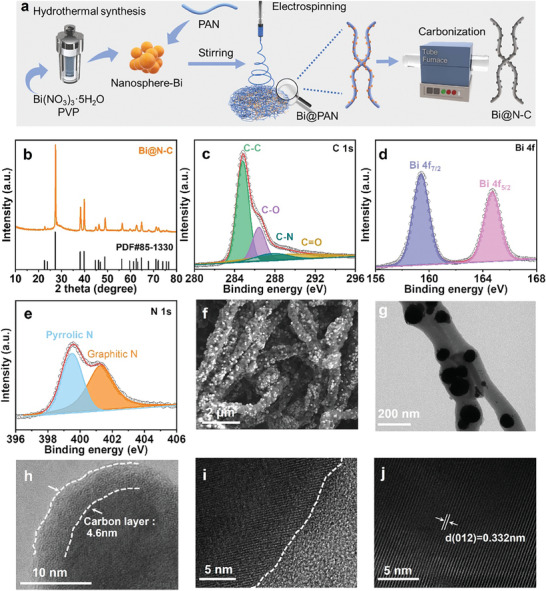
a) Schematic diagram of the preparation process of Bi@N‐C. b) XRD pattern, High‐resolution XPS spectrum of (c) C1s, (d) Bi 4f, and (e) N1s of Bi@N‐C. f) SEM, g) TEM image of Bi@N‐C. h–j) High‐resolution TEM images of Bi@N‐C.

**Figure** **1**a schematically illustrates the synthesis route of Bi@N‐C. First, the uniform Bi nanoparticles were synthesized on the basis of the reported solvothermal method,^[^ ^11^ ^]^ which were an average of ≈ 150 nm (Figure S1, Supporting Information). Then, the Bi nanoparticles mixed with polyacrylonitrile (PAN) were dispersed in N, N‐Dimethylformamide (DMF), and the disperser was homogeneously electrospinning into Bi@PAN (Figure S2, Supporting Information). Then, Bi@N‐C hybrid was obtained by carbonizing Bi@PAN in the Ar atmosphere. XRD, Raman, and XPS were characterized to confirm the crystal structure and chemical valences of Bi@N‐C. In Figure 1b, the diffraction peaks of Bi@N‐C are depicted, exhibiting a good match with the standard Bi pattern (JCPDS Card No. 85–1330). Besides, no additional diffraction peaks are observed in the XRD pattern. The signals of the carbon layer cannot be characterized by XRD due to the amorphous feature. However, the presence of carbon was confirmed by the Raman spectrum. As shown in Figure S3 (Supporting Information), the two typical peaks at ≈ 1340 and 1590 cm^−1^, which are corresponding to the D peak of amorphous carbon and defects and the G peak of graphite‐like structure.^[^ ^12^ ^]^ The *I_D_/I_G_* ratio of Bi@N‐C is ≈ 1.30, reflecting the abundant defects and disorder in N‐doped carbon. Figure S4 (Supporting Information) shows a typical XPS survey spectrum of Bi@N‐C, which indicates this hybrid consists of Bi, C and N. The peaks with the binding energy of 284.8, 286.2, 287.9, and 289.4 eV in the high‐resolution C 1s spectrum are assigned to the C─C, C─O, C─N, and C═O bonds, respectively (Figure 1c).^[^ ^12^ ^]^ The peaks observed at 159.3 and 164.7 eV in the Bi 4f spectrum originate from the Bi 4f_7/2_ and Bi 4f_5/2_, respectively (Figure 1d).^[^ ^13^ ^]^ The two peaks at 399.5 and 401.3 eV in the high‐resolution XPS of N 1s spectrum can be fitted to pyrrolic N and graphitic N, respectively (Figure 1e). Besides, the N content on the surface of the Bi@N‐C hybrid is determined to be 12.01 at%, a factor contributing to enhanced electroconductivity of the composite and the provision of generous electrochemical active sites in this hybrid (Table S1, Supporting Information). Figure 1f shows the scanning electron microscope (SEM) images of the Bi@N‐C hybrid, which indicate the uniform dispersion of Bi nanoparticles in the N‐doped carbon nanowires. In addition, the transmission electron microscopy (TEM) image confirms the uniformity of Bi nanoparticles on nanowires (Figure 1g), which is concordant with the SEM images. High‐resolution TEM (HRTEM) images of Bi@N‐C clearly reveal the Bi nanoparticles are embedded into disordered lattice carbon nanowires and the thickness of the outer carbon layer is ≈ 4.6 nm (Figure 1h,i). In the process of sodiation/disodiation, N‐doped carbon can not only promote electron/ion transfer and improve electron conductivity, but also effectively relieve the volume expansion. In the atomic‐resolution TEM image of the Bi@N‐C hybrid, the lattice fringes with an interlayer spacing of 0.332 nm corresponding to (012) plane of Bi (Figure 1j). STEM and the corresponding energy‐dispersive X‐ray spectrum (EDX) elemental mappings indicate the uniform distribution of Bi nanoparticles and carbon layers in Bi@N‐C (Figure S5, Supporting Information). Besides, from the thermogravimetric analysis (TGA), the Bi content in Bi@N‐C is 60.4 wt.% (Figure S6, Supporting Information).

**Figure 2:**
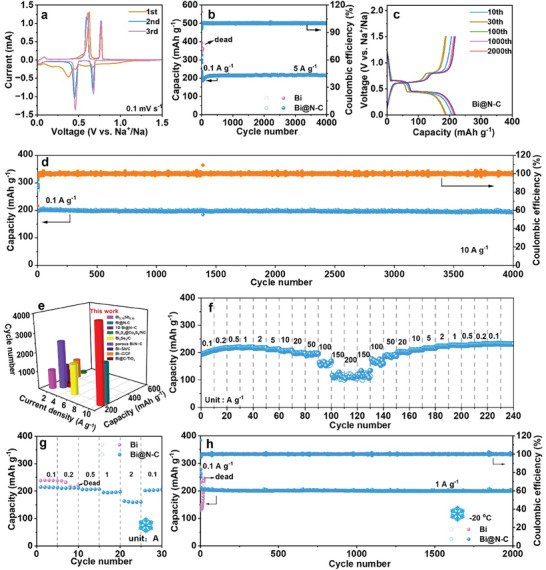
Electrochemical performance of the Bi@N‐C electrode. a) CV curves of Bi@N‐C at a scan rate of 0.1 mV s^−1^. b) Cycling performance and c) charge/discharge profiles of Bi and Bi@N‐C at a current density of 5 A g^−1^. d) Cycling performance of Bi@N‐C at 10 A g^−1^. e) The cycling performance comparison with reported Bi‐based anode materials for SIBs. f) Rate performance of Bi@N‐C. g) Rate performance and h) cycling performance of Bi and Bi@N‐C at −20 °C.

The sodium storage performance of the Bi@N‐C was characterized in half cells to validate its commercial potential as an anode in SIBs. **Figure** **2**a shows the Cyclic voltammetry (CV) curves of Bi@N‐C at a scan rate of 0.1 mV s^−1^ in the voltage range of 0.01 to 1.5 V (vs Na^+^/Na). In the first cathodic scan, the two cathode peaks at 0.67 and 0.36 V are assigned to the sodiation process of Bi→NaBi→Na_3_Bi, and the two oxidation peaks centered at 0.61 and 0.76 V are attributed to the desodiation reactions of Na_3_Bi→NaBi→Bi. After the first cycle, the overlap of the redox peaks indicates the good reversibility of Bi@N‐C. The process of sodiation/desodiation is consistent with Bi microparticles (Bi) (Figures S7 and S8, Supporting Information). Figure S9 (Supporting Information) depicts the charge and discharge curves of Bi@N‐C and Bi at 0.1 A g^−1^, and all the charge/discharge platforms are consistent well with the redox peaks of CV curves. The corresponding cycling performances of Bi@N‐C and Bi are shown in Figure 2b. Bi@N‐C presents a stable cycling up to 4000 cycles at 5 A g^−1^ with a capacity retention of 94.3%, while the cycling life of Bi anode is only 31 cycles. Figure 2c and Figure S10 (Supporting Information) show the charge/discharge curves at different cycles in the cycling process, the capacity of Bi@N‐C tends to be stable after the initial activation stage, contrasting with Bi electrode, which fails after 31 cycles. Remarkably, the Bi@N‐C maintains outstanding cycling performance even at large current density (Figure 2d). At the current density of 10 A g^−1^, the Bi@N‐C electrode delivers a capacity of 194.3 mAh g^−1^ after 4000 cycles with a capacity retention of 95.3%, which is an outstanding cycling performance for Bi‐based anode materials to date (Figure 2e).^[^ ^14^ ^]^ As shown in Figure 2f, the rate performance of Bi@N‐C further affirms its excellent electrochemical performance. The reversible capacity of Bi@N‐C is 208.0, 213.1, 218.4, 218.5, 217.1, 212.3, 206.5, 199.1, 191.1, 152.2, 119.5, and 110.0 mAh g^−1^ at the current density of 0.1, 0.2, 0.5, 1, 2, 5, 10, 20, 50, 100, 150, and 200 A g^−1^. At the current density of 200 A g^−1^, the capacity retention is 52.9% compared with the capacity at 0.1 A g^−1^ (Figure S11, Supporting Information). Furthermore, the low‐temperature electrochemical performance of Bi@N‐C was tested. As illustrated in Figure 2g, Bi@N‐C shows the capacity of 215.9, 213.8, 208.5, 197.5, and 161.2 mAh g^−1^ at the current density of 0.1, 0.2, 0.5, 1, 2 A g^−1^ at −20 °C, while Bi electrode fails at the current density of 0.2 A g^−1^. It is shown in Figure 2h and Figure S12 (Supporting Information) that the Bi electrode just can cycle for 24 cycles at the current density of 1 A g^−1^ at −20 °C, while the Bi@N‐C can cycle for over 2000 cycles with a high capacity retention of 97.6%. The exceptional electrochemical performance is attributed to the N‐doped carbon, which not only guarantees the rapid kinetics but also suppresses the stress caused by volume expansion during the alloying/dealloying process.

**Figure 3:**
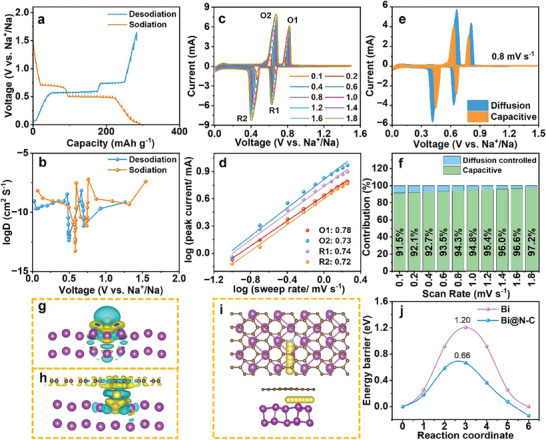
a) GITT profiles of the sodiation/desodiation process and b) the sodium ion diffusion coefficient as a function of the state of sodiation/desodiation process of Bi@N‐C electrode. c) CV curves at various scan rates, d) log (i) versus log (ν) plots for the cathodic/anodic peaks of Bi@N‐C. Capacitive contributions of Bi@N‐C at e) 0.8 mV s^−1^ and f) different scan rates. The charge density difference of g) Bi and h) Bi/N‐graphene, i) the diagrams of sodium migration path on the Bi/N‐graphene surface. j) The migration energy barriers on the Bi and Bi/N‐graphene surface.

The electrochemical kinetic behavior was investigated to verify the excellent sodium storage performance of Bi@N‐C. The galvanostatic intermittent titration technique (GITT) was tested to investigate the electrochemical kinetics of Bi@N‐C (**Figure** **3**a). The GITT data was collected at a current density of 50 mA g^−1^ for 10 min interspersed with a rest interval of 0.5 h. The relationship between the ion diffusion coefficient of the electrode and the voltage can be determined by the following Equation (1):

(1) $$D\;=\frac{4}{\pi \tau }\;{\left(\frac{nV_{m}}{S}\right)}^{2}{\left(\frac{\Delta E_{s}}{\Delta E_{\tau }}\right)}^{2}$$

where τ represents the current pulse time, *V_m_* denotes the molar volume of active materials, S is the area of the electrode, Δ*E_s_* indicates the change in steady‐state voltage during the potential step of the plateau, and Δ*E*_τ_ is the total voltage change of the battery during the current pulse.^[^ ^15^ ^]^ The diffusion coefficients are relative to the electrochemical process, and the minimal D is found at the voltage plateaus during the sodiation/desodiation process (Figure 3b), which indicates a gradual sodiation/desodiation process is based on the electrochemical reaction. As shown in Figure 3c, CV curves at various sweep rates from 0.1 to 1.8 mV s^−1^ were tested to verify the kinetics process of the Bi@N‐C electrode. The relationship of the obtained peak current (i, mA) and scan rate (v, mV s^−1^) is followed by the formula: i = av^b^. b is closely associated with the character of the electrochemical process, tends to be 0.5 for the diffusion‐controlled process and ≈ 1.0 for the surface capacitance‐based process in the reported literature.^[^ ^16^ ^]^ As linearly fitted of the two couple of redox peaks (R1/O1, R2/O2) (Figure 3d), the b values of two anodic peaks and two cathodic peaks are 0.74, 0.72, 0.78, and 0.73, respectively, indicating the sodium storage process is a mixture of diffusion‐controlled process and surface capacitance‐based process battery behavior. Moreover, the two couple redox peaks correspond to the alloying/dealloying process, it can be concluded that the rate of alloying is comparable to the case of dealloying.^[^ ^10b^ ^]^ The total capacitance contribution to the current response was calculated based on the equation: i = k_1_v+k_2_v^1/2^, where i is the anodic or cathodic current, v is the scan rate, k_1,_ and k_2_ are adjustable constants in this equation.^[^ ^17^ ^]^ According to the integration of the CV curves, the pseudo‐capacitance ratio is ≈ 94.3% at the scan rate of 0.8 mV s^−1^ (Figure 3e). Figure 3f summarizes the proportion of pseudo‐capacitance contribution gradually increases from 91.5 to 97.2% across different scanning rates raging 0.1 to 1.8 mV s^−1^. Such high pseudo‐capacitance contribution leads to rapid kinetics during the charge/discharge process. The sodium migration based on DFT calculation was calculated to confirm the enhanced kinetics of Bi@N‐C, and an N‐doped graphene monolayer was applied to represent the carbon layer for a simplified model for considering the complexity of the actual carbon layer coated on the Bi nanoparticles. As shown in Figure 3g,h, the N‐graphite coating on the Bi surface can improve the conductivity and redistribute the electron density around the Na. The side view and top view of the Na‐ion migration pathways on the Bi/N‐graphene interface and the Bi surface are shown in Figure 3i and Figure S13 (Supporting Information). Moreover, the Na‐ion migration energy barriers in Figure 3j shows the migration energy barrier of Bi@N‐C (0.66 eV) is much lower than Bi (1.20 eV). The lower migration energy barrier indicates that diffusion is easier to occur on the Bi/N‐graphene surface than on the Bi surface, indicating the positive effects of N‐graphene layers.

**Figure 4:**
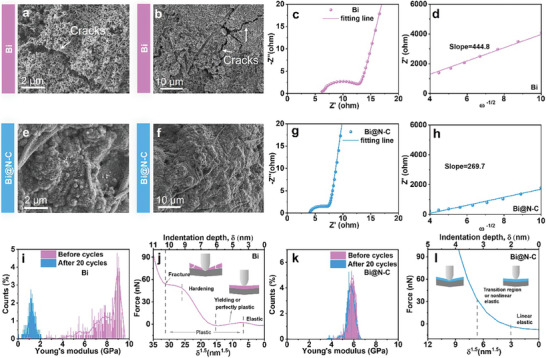
a,b) SEM images of Bi electrode after 20 cycles, c) EIS spectra, and d) plots of z’ versus ω^−1/2^ in the low‐frequency region. e,f) SEM images of Bi@N‐C electrode after 20 cycles, g) EIS spectra and h) plots of z’ versus ω^−1/2^ in the low‐frequency region. Young's modulus of i) Bi and k) Bi@N‐C before cycling and after 20 cycles. Force spectroscopy of j) Bi and k) Bi@N‐C electrode after 20 cycles.

For a deeper level of understanding the factors contributing to the excellent electrochemical performance of Bi@N‐C, SEM, EIS, and AFM tests were conducted. Before cycles, both Bi and Bi@N‐C are uniformly dispersed in the electrode. (Figures S14 and S15, Supporting Information). It can be seen that there are a number of cracks on the surface of the Bi electrode after 20 cycles (**Figure** **4**a,b), which agrees well with the degraded performance. In contrast, there is no notable rupture in the Bi@N‐C electrode (Figure 4e,f; Figure S16, Supporting Information), the result indicates that the carbon layer can provide an effective buffer to mitigate the severe volume change of Bi. As shown in Figure 4c,d,g,h, the smaller charge transfer impedance (R_ct_) and lower slope observed from the EIS spectra, which indicate the better charge transfer and ion diffusion kinetics of Bi@N‐C benefitted from the ion/electron transmission channels of N‐dopped carbon layers. The superior electrochemical performance of Bi@N‐C must correspond to the dissipation of stress in electrode, which can be characterized by AFM (Figure 4i–l; Figure S17, Supporting Information). As shown in Figure 4i, the average Young's modulus of Bi electrode is 9.0 GPa before cycling, and the Young's modulus goes down to 1.2 GPa after 20 cycles. It is very different for Bi@N‐C, the Young's modulus of before cycling and after 20 cycles are almost same, with the values of 5.8 and 5.5 GPa respectively (Figure 4k).

The response profiles of the Bi electrode can be categorized into the following stages: i) an elastic region, characterized by a nearly linear correlation between force and δ^1.5^ (δ is the indentation depth); ii) a yielding or nonlinear elastic region, where the force is almost quasi‐constant; iii) a hardening region, marked by a non‐linear increase in force and iv) a fracture region, where the force decreases gradually and ruptures appear on the electrode. In contrast, the response profiles of Bi@N‐C are totally different, with only a linear elastic region and a slope‐changing region or the non‐linear elastic region.^[^ ^18^ ^]^ These differences indicate that electrode ruptures are avoided in Bi@N‐C electrode, which leads to an extended life span. The superior mechanical property of Bi@N‐C electrode implies that the Bi@N‐C with an N‐dopped carbon layer can effectively resist the big change of structural stress and maintain the electrode integrity.

**Figure 5:**
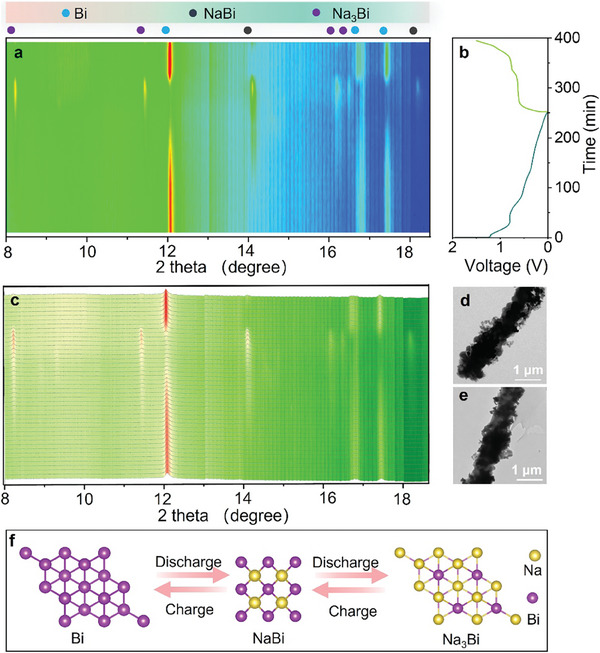
a) Contour plot, b) galvanostatic discharge/charge profiles at the current density of 0.1 A g^−1^, c) synchrotron in‐situ XRD patterns of Bi@N‐C. TEM images of Bi@N‐C after d) 20 cycles and e) 50 cycles. f) schematic diagram of alloying/dealloying process of Bi@N‐C.

To further investigate the sodium storage mechanism of Bi@N‐C, synchrotron in situ XRD was employed in the initial cycle (**Figure** **5**). Figure 5a,c presents the collected XRD patterns, and Figure 5b shows the discharge/charge profiles of Bi@N‐C in the first cycle at the current density of 0.1 A g^−1^. Before the process of discharging, the peaks at 12.0^°^, 16.7^°,^ and 17.4^°^ are well corresponded with Bi (Figure 5a). During the sodiation process, the peaks associated with Bi gradually diminish within the voltage range of 1.5‐0.80 V. In the first voltage platform of 0.80–0.54 V, the Bi peaks fade away while the peaks of NaBi at 14.1^°^, and 18.2^°^ gradually strengthen, indicating the alloying reaction forming the NaBi phase. As sodiation progresses into the second voltage window of 0.54‐0.01 V, the peaks at 8.2^°^, 11.4^°^, 16.2^°^, and 16.5^°^ of Na_3_Bi emerge, while peaks of NaBi diminish. The peaks of Bi reappear and persist during charging, which is associated with the dealloying of Na_3_Bi. In the full‐charged electrode, only peaks of Bi appear in the XRD pattern, which indicates the good reversibility of Bi@N‐C. The structure of Bi encapsulated in an N‐doped carbon nanowire can be observed in the Bi@N‐C electrode after 20 cycles (Figure 5d) and 50 cycles (Figure 5e). Based on the above discussion, the alloying/dealloying process of Bi@N‐C in SIBs is summarized in Figure 5f.

**Figure 6:**
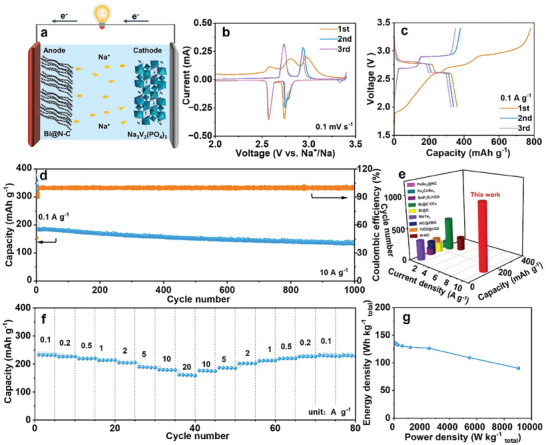
a) Schematic diagram of discharge process of Bi@N‐C||NVP@C full cell. b) CV curves, c) charge/discharge profiles, d) cycling performance of Bi@N‐C||NVP@C full cell. e) Comparison of the electrochemical performance of the reported full cells with ours. f) Rate capacity and g) Ragone plots of Bi@N‐C||NVP@C full cell.

Owning to the excellent electrochemical performance of Bi@N‐C in half cell, the full cell with Bi@N‐C as anode, and the home‐made Na_3_V_2_(PO_4_)_3_@C (NVP@C).^[^ ^19^ ^]^ (Figure S18, Supporting Information) as cathode was assembled to explore the practical application in SIBs (**Figure** **6**a). In Figure 6b, there are two pairs of the redox peaks at 2.5/2.7 V, and 2.7/2.9 V are corresponding to the insertion/deinsertion process in the CV curves of the full cell. The overlapped CV curves after the first cycle indicates the excellent reversibility of the full cell. Figure 6c shows the charge/discharge profiles of the full cell at 0.1 A g^−1^ in the voltage of 2.0–3.4 V, and the platforms are consistent with the CV curves. Figure 6d shows the cycling performance of the full cell at the current density of 10 A g^−1^, there is a capacity of 136.1 mAh g^−1^ after 1000 cycles, corresponding to a capacity retention of 73.0%. Figure 6e shows the comparison of the cycling performance of full cells, Bi@N‐C||NVP@C shows the high capacity at high current density and long cycling life compared with the reported works.^[^ ^5^, ^20^ ^]^ Figure 6f shows the rate capacity of the full cell, Bi@N‐C||NVP@C can deliver a high capacity of 161.0 mAh g^−1^ even at high current density of 20 A g^−1^. Figure 6g shows the Ragone plots of Bi@N‐C||NVP@C full cell, and the full cell can deliver an energy density up to ≈ 136 Wh kg^−1^. Even at 9034.0 W kg^−1^, there is still an energy density of 90.4 Wh kg^−1^. The excellent cycling performance and good rate capacity of Bi@N‐C||NVP@C indicates the potential application of Bi@N‐C.

## 3 Conclusion

In summary, we have successfully fabricated the Bi@N‐C via facile electrospinning and carbonization. Ex‐situ SEM, synchrotron in‐situ XRD, AFM, and DFT calculations show the remarkable stability and improved mechanical capacity of Bi@N‐C. The N‐doped carbon layer‐coated Bi nanoparticles serve as an effective buffer mitigating volume changes during alloying/dealloying and improves the electrons/ions conductivity for rate capacity. Benefited from the structure of composite, Bi@N‐C can deliver a capacity of 197.7 mAh g^−1^ after 4000 cycles at 10 A g^−1^ with a capacity retention of 95.3% and 110.0 mAh g^−1^ even at 200 A g^−1^. Bi@N‐C can stably cycle for over 2000 cycles at −20 °C with a high capacity retention of 97.6%. Furthermore, the full cell composed of Bi@N‐C and NVP@C can deliver 136.1 mAh g^−1^ after 1000 cycles at the current density of 10 A g^−1^, and an energy density of 90.4 Wh kg^−1^ even at 9034.0 W kg^−1^. The excellent cycling capacity and rate performance of Bi@N‐C indicates the practical application of alloying‐based anodes.

## 4 Experimental Section

### Synthesis of Bi@N‐C

Bi nanoparticles were fabricated according to the reported work.^[^ ^12^ ^]^ Initially, 0.364 g of Bi(NO_3_)_3_•5H_2_O (99.0%, Macklin) and 0.6 g of polyvinyl pyrrolidone (PVP, average molecular weight = 10 000, Aladdin) were dissolved in a mixture of 10 ml of 1 m HNO_3_ (AR, Sinopharm) aqueous solution and 50 ml of ethanol (99.7%, J&K Scientific). The resulting mixture was transferred to a 100 ml polytetrafluoroethylene lined stainless steel autoclave and reacted at 150 °C for 12 h. Afterward, the sample was centrifuged, separated, washed with deionized water and ethanol, and finally dried under vacuum at 70 °C for 10 h. The dried sample is Bi nanoparticles. Then, 0.8 g of Bi nanoparticles were dispersed in N, N‐Dimethylformamide (DMF, 99.8%, J&K Scientific), and 0.4 g of polyacrylonitrile (PAN, average molecular weight ≈ 149 000, Aladdin) was added into the dispersion. The mixture was stirred at 60 °C for 9 h. Then the resulting solution was electrospun at the voltage of 17 kV and the speed of 1 ml h^−1^, with the collection distance of 12 cm. Subsequently, the spun product was annealed at 600 °C under argon gas for 2 h.

### Materials Characterization

Scanning electron microscope (SEM) images were acquired using a filed‐emission scanning microscope (FESEM, ZEISS Geminisem 300, Germany). X‐ray powder diffraction (XRD) patterns were recorded on a diffractometer (Brucker D8 Advanced, Germany) with the monochromatic Cu Kα line as the radiation (λ = 1.5418 Å). Raman spectroscopy was characterized using a micro‐Raman spectrometer (Horiba JY Lab RAM HD88, Japan) with excitation at 532 nm at room temperature. X‐ray photoelectron spectroscopy (XPS) was collected using Al‐Kα radiation (hv = 1486.6 eV) as the X‐ray source on a spectrometer (ESCALAB 250Xi^+^, Thermo Scientific, USA). The high‐resolution XPS spectra were calibrated by the binding energy of C 1s as 284.8 eV. The thermogravimetric analysis (TGA) was measured in the air using a thermal analyzer (Mettler Toledo TGA/SDTA 851, Switzerland) from room temperature to 800 °C with a heating rate of 10 °C min^−1^. Transmission electron microscope (TEM) images were recorded on a transmission electron microscope (Hitachi 7700, Japan). High‐resolution transmission TEM (HRTEM) images were measured on a transmission electron microscope (STEM, FEI Talos F200X, USA). Force spectroscopy measurements (AFM) were conducted on a scanning scope (Brucker Bioscope Resolve, Germany) via the tapping mode with the probe (RTESPA‐525, Germany). The testing size was collected over 5 × 5 µm^2^ at a resolution of 256 × 256 pixels.

### Electrochemical Measurements

The slurry of anode was prepared by combining active materials, acetylene black (AB, Dodochem) and carboxymethyl cellulose (CMCNa, Dodochem) with a weight ratio of 7:2:1. Spread the slurry onto a copper foil and dry overnight at 60 °C. Subsequently, the foil was cut into discs with a diameter of 10 mm and the active materials mass loading of 1.2–1.4 mg cm^−2^. These obtained discs as working electrodes were assembled into 2025‐type coin cells in the Ar‐filled glove box (Mikrouna, Super 1220/750/900) with Na foils as working and counter electrodes with glass microfibers (Whatman GF/F) as separators and 1 m NaPF_6_ in diethylene glycol dimethyl ether electrolyte (Dodochem) as the electrolyte. The electrochemical performance of half cells was tested on multichannel battery‐testing systems (Land CT2001A, China) within 0.01–1.5 V (vs Na^+^/Na). The low‐temperature electrochemical performance was measured in an incubator (Yiheng Scientific Instrument, China) at −20 °C. CV curves were measured on an electrochemical workstation (CHI 760D, Chenhua Instruments, China) in the voltage of 0.01–5 V at a scanning rate of 0.1 mV s^−1^. Electrochemical impedance spectra (EIS) were recorded on an electrochemical workstation (Autolab PGSTAT 302N, Switzerland) in the frequency range of 100 KHz to 0.01 Hz.

For full cells, Na_3_V_2_(PO_4_)_3_@C (NVP@C) was fabricated by the reported work.^[^ ^18^ ^]^ NVP@C, AB, and polyvinylidene difluoride (PVdF, Dodochem) were mixed by a mass ratio of 8:1:1, then the slurry was spread onto a clean aluminum foil after ball milling for 2 h at 350 rpm, the foils were dried at 100 °C for 10 h. The capacity of the anode and cathode was ≈1:1.2.

### Theoretical Calculation

Density functional theory (DFT) calculations were carried out using the Vienna ab initio simulation package (VASP) with the method of the projector augmented wave (PAW).^[^ ^21^ ^]^ The Perdew‐Brucker‐Ernzerhof (PBE) exchange‐correlation function was doped for generalized gradient approximation (GGA) correction.^[^ ^22^ ^]^ The wave function is expanded at an energy cutoff of 400 eV. To avoid periodic repetition, a Monkhorst‐Pack grid of 1 × 2 × 1 points is set for the Bi(012) surface, and a vacuum layer of ≈ 15 Å is employed to eliminate interactions between periodic slabs. The climbing‐image nudged elastic band (CINEB) method is utilized to derive the transition state for Na ion dissociation.

## Conflict of Interest

The authors declare no conflict of interest.

## Supporting information

Supporting Information

## Data Availability

The data that support the findings of this study are available in the supplementary material of this article.
