# Supplementary material for: Bi Nanospheres Embedded in N‐Doped Carbon Nanowires Facilitate Ultrafast and Ultrastable Sodium Storage
Source: Adv Sci (Weinh). 2024 May 2;11(28):2401730. doi: 10.1002/advs.202401730 (PMC11267272; doi:10.1002/advs.202401730)
Supplement: Supplementary file 1 — Supporting Information [file ADVS-11-2401730-s001.pdf]

ADVANCED SCIENCE
Open Access

Supporting Information

for *Adv. Sci.*, DOI 10.1002/advs.202401730

Bi Nanospheres Embedded in N-Doped Carbon Nanowires Facilitate Ultrafast and Ultrastable Sodium Storage

Qian Yao, Cheng Zheng, Kejun Liu, Mingyue Wang, Jinmei Song, Lifeng Cui, Di Huang, Nana Wang, Shi Xue Dou, Zhongchao Bai* and Jian Yang**

Supplementary Material for

Bi Nanospheres Embedded in N-Doped Carbon Nanowires Facilitate Ultrafast and Ultrastable Sodium Storage

Qian Yao, Cheng Zheng, Kejun Liu, Mingyue Wang, Jinmei Song, Lifeng Cui, Di Huang, Nana Wang, Shixue Dou, Zhongchao Bai, Jian Yang

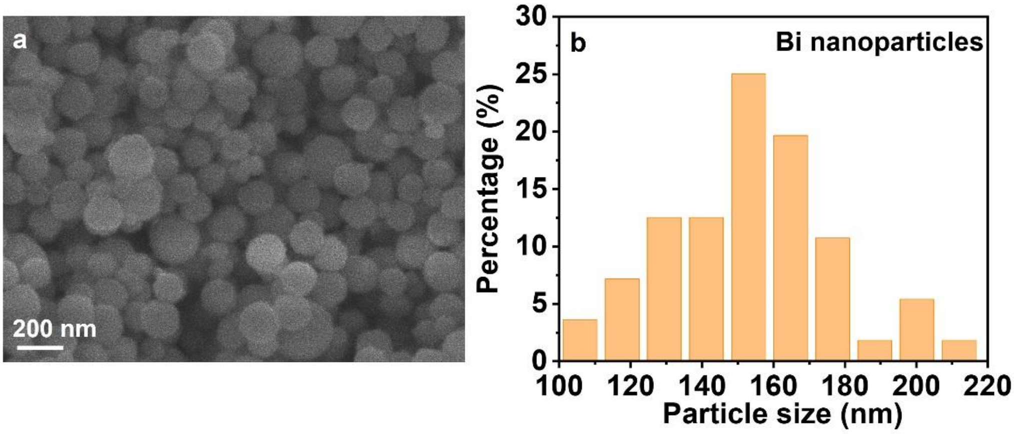

Figure S1. (a) SEM images of Bi nanoparticles and (b) statistics of Bi nanoparticles.

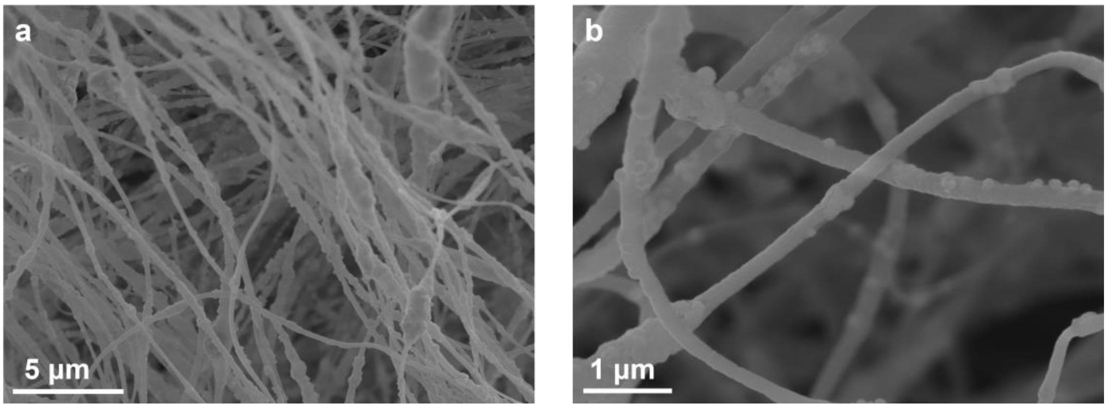

Figure S2. SEM images of Bi@PAN.

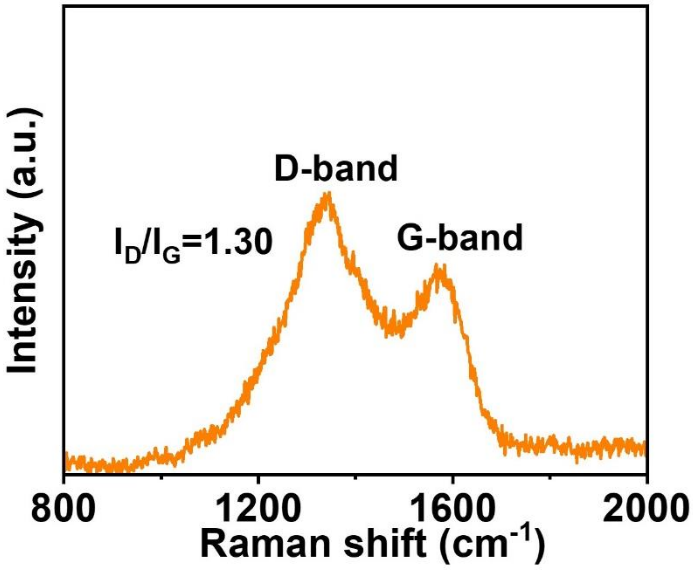

Figure S3. Raman spectrum of Bi@N-C.

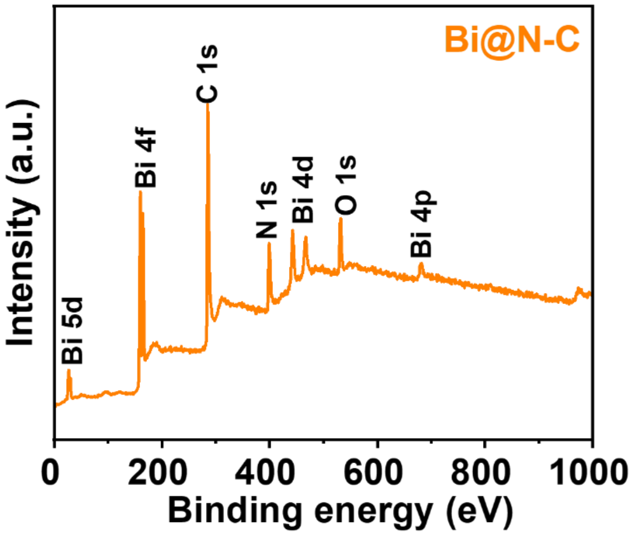

Figure S4. XPS survey spectrum of Bi@N-C.

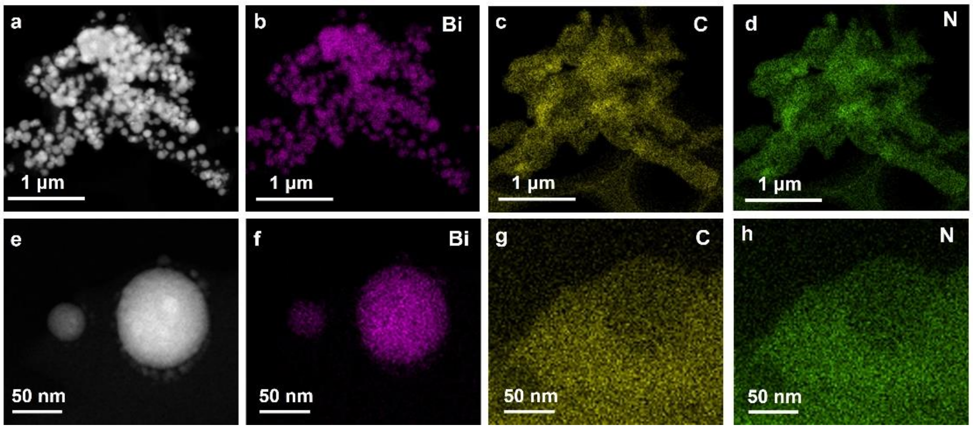

Figure S5. (a, e) The dark-field scanning TEM (DF-STEM) and (b-d, f-h) corresponding elemental mapping images of Bi@N-C.

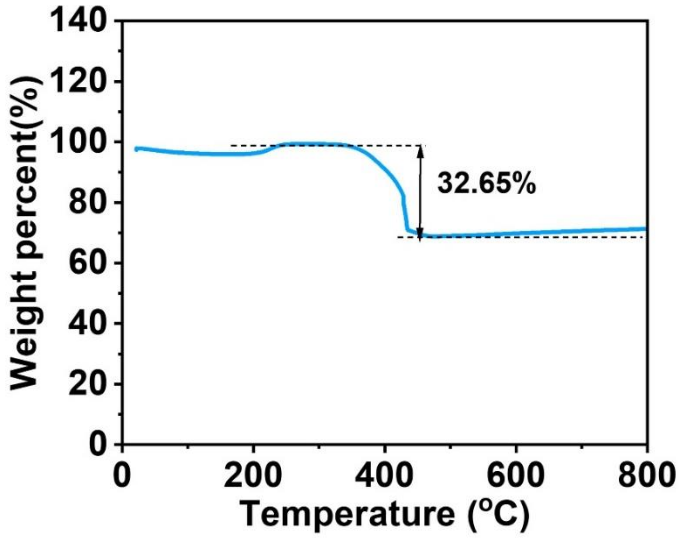

Figure S6. TGA curve of Bi@N-C.

Based on the previous reports, the distinct weight loss is associated with the following reaction in the region of 200 – 450 °C.

$$4\ Bi + 3\ O_2 = 2\ Bi_2O_3$$

$$C + O_2 = CO_2$$

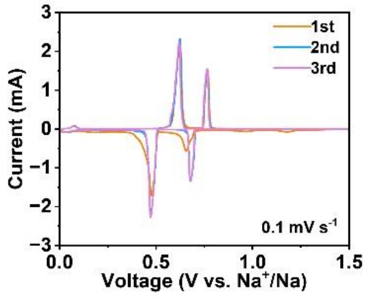

Figure S7. CV curves of Bi at a scan rate of 0.1 mV s^{-1}.

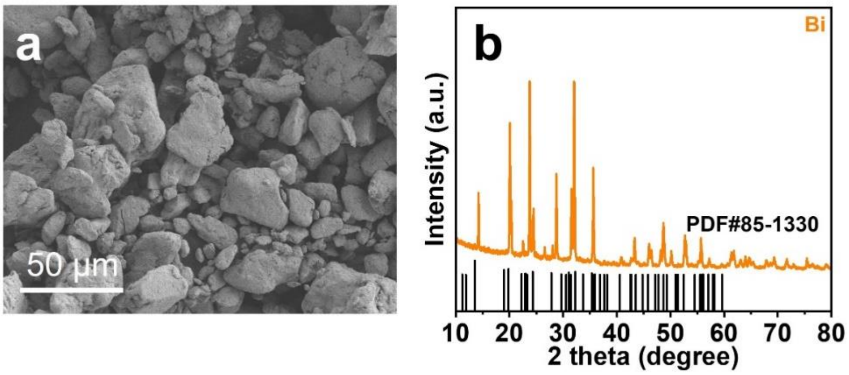

Figure S8. (a) SEM image, (b) XRD pattern of Bi microparticles.

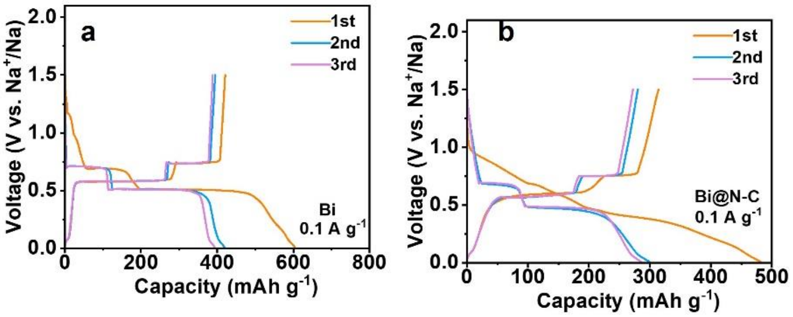

Figure S9. The first three charge/discharge profiles of (a) Bi and (b) Bi@N-C at a current density of 0.1 A g^{-1}.

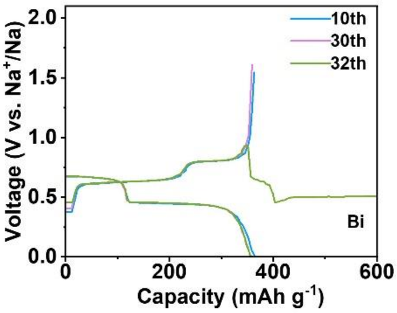

Figure S10. Charge/discharge profiles of Bi at a current density of 5 A g^{-1}.

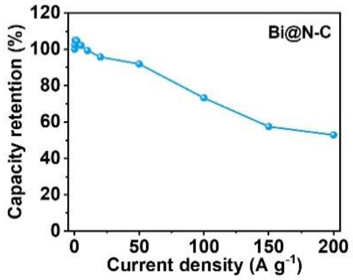

Figure S11. The capacity retention of Bi@N-C at different current density.

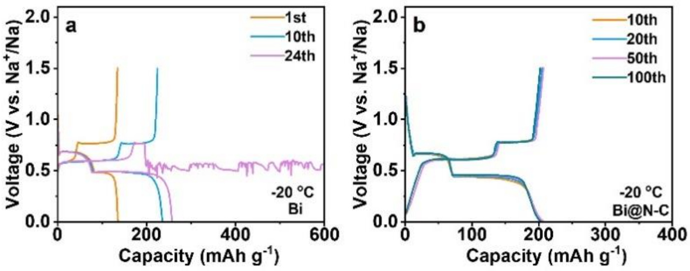

Figure S12. Charge/discharge profiles of (a) Bi and (b) Bi@N-C at different cycles at -20 °C.

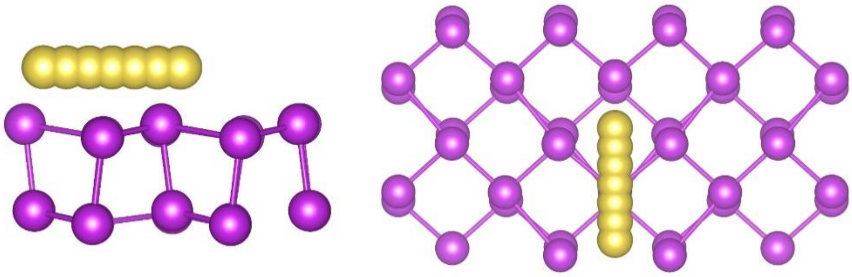

Figure S13. The diagrams of sodium migration path on the Bi surface.

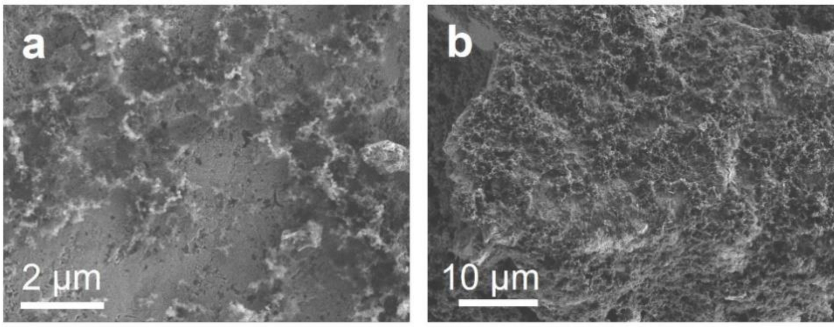

Figure S14. SEM images of Bi before cycling.

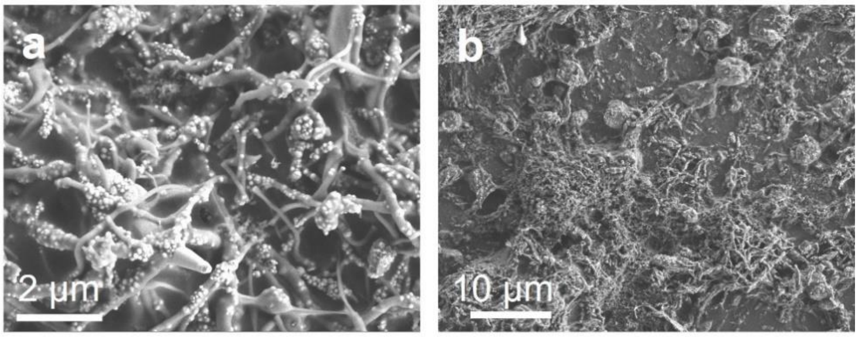

Figure S15. SEM images of Bi@N-C before cycling.

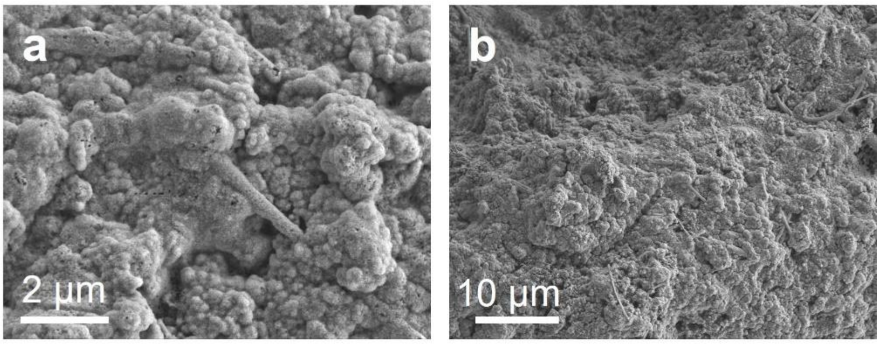

Figure S16. SEM images of Bi@N-C after 200 cycles.

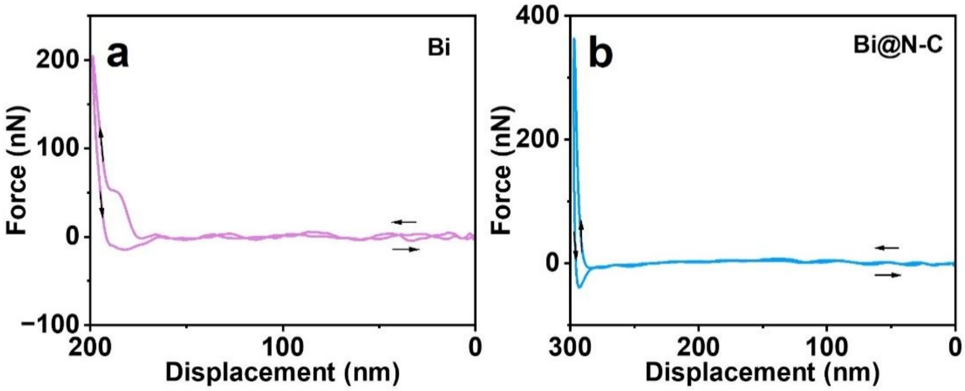

Figure S17. Force responses of the (a) Bi and (b) Bi@N-C electrodes after 20 cycles.

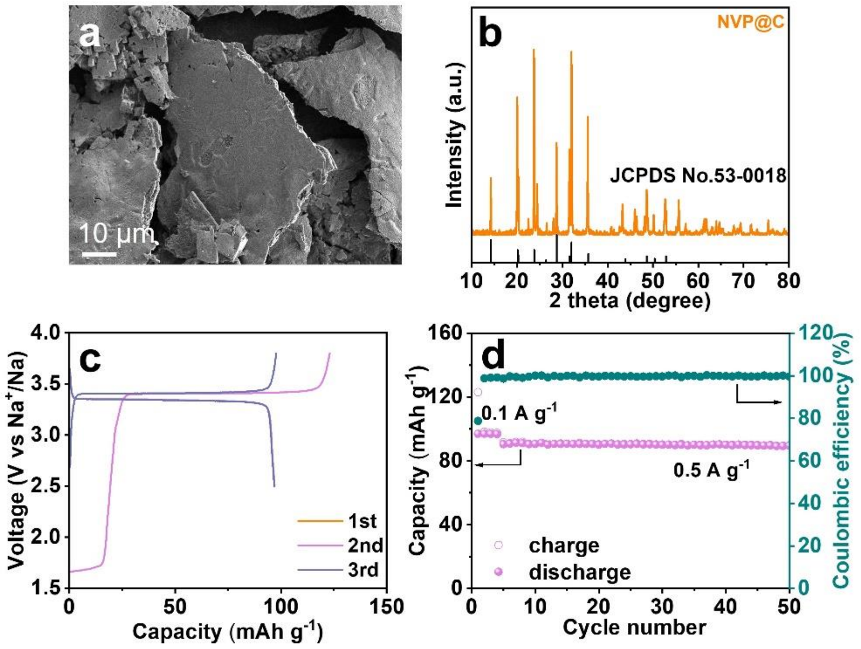

Figure S18. (a) SEM image, (b) XRD pattern, (c) charge/discharge profiles and (d) cycling performance of NVP@C.

Table S1. The proportion of different elements from the XPS spectra.

Element	C	N	O	Bi
Atomic/%	75.09	12.01	5.41	7.49
